# Exploring Kainic Acid-Induced Alterations in Circular Tripartite Networks with Advanced Analysis Tools

**DOI:** 10.1523/ENEURO.0035-24.2024

**Published:** 2024-07-26

**Authors:** Andrey Vinogradov, Emre Fikret Kapucu, Susanna Narkilahti

**Affiliations:** Faculty of Medicine and Health Technology, Tampere University, Arvo Ylpön katu 34, Tampere 33520, Finland

**Keywords:** brain-on-a-chip, burst, microelectrode array, neuronal connectivity, neuronal culture, signal processing

## Abstract

Brain activity implies the orchestrated functioning of interconnected brain regions. Typical in vitro models aim to mimic the brain using single human pluripotent stem cell-derived neuronal networks. However, the field is constantly evolving to model brain functions more accurately through the use of new paradigms, e.g., brain-on-a-chip models with compartmentalized structures and integrated sensors. These methods create novel data requiring more complex analysis approaches. The previously introduced circular tripartite network concept models the connectivity between spatially diverse neuronal structures. The model consists of a microfluidic device allowing axonal connectivity between separated neuronal networks with an embedded microelectrode array to record both local and global electrophysiological activity patterns in the closed circuitry. The existing tools are suboptimal for the analysis of the data produced with this model. Here, we introduce advanced tools for synchronization and functional connectivity assessment. We used our custom-designed analysis to assess the interrelations between the kainic acid (KA)-exposed proximal compartment and its nonexposed distal neighbors before and after KA. Novel multilevel circuitry bursting patterns were detected and analyzed in parallel with the inter- and intracompartmental functional connectivity. The effect of KA on the proximal compartment was captured, and the spread of this effect to the nonexposed distal compartments was revealed. KA induced divergent changes in bursting behaviors, which may be explained by distinct baseline activity and varied intra- and intercompartmental connectivity strengths. The circular tripartite network concept combined with our developed analysis advances importantly both face and construct validity in modeling human epilepsy in vitro.

## Graphical abstract

**Figure:**
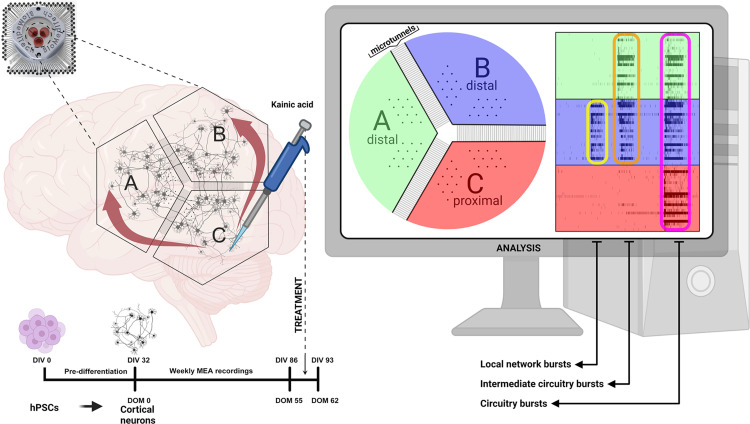
Visual Abstract

## Significance Statement

The scope of this work covers the analysis of in vitro electrophysiological recordings of neuronal populations derived from human pluripotent stem cells. The recordings were obtained using a state-of-the-art compartmentalized microfluidic device with an embedded microelectrode array (MEA) designed for in vitro modeling of a circular tripartite network of three spatially divergent brain areas. Neuronal signals were processed to explore the functioning of the neuronal networks. Custom MEA data analysis tools were developed to assess features of the formed networks, including circuitry-level synchronization and functional connectivity. The circular tripartite network model combined with an integrated analysis pipeline provides an opportunity for modeling neurological disorders and screening pharmacological compounds in vitro in a more validated manner.

## Introduction

Neurological disorders cause ∼10 million deaths annually and drastically reduce the quality of life of surviving patients (Ding et al., 2022)*.* Epilepsy alone affects 65 million people worldwide, one-third of whom are not achieving proper seizure control with the currently available treatments (Golyala and Kwan, 2017). The complexity of the human brain hinders the development of novel treatments. Conventional neuroimaging tools such as EEG, MEG (Coolen et al., 2018), and fMRI (Centeno and Carmichael, 2014; Lewis et al., 2016) provide researchers with macrolevel brain functional data but have limited spatial and temporal resolution (Depannemaecker et al., 2022; Ihle et al., 2022). On the other hand, microelectrode array (MEA) recordings enable the collection of information from hundreds of neurons, e.g., in epileptic patients (Peyrache et al., 2012; Dehghani et al., 2016; Paulk et al., 2022). There are obvious limitations to clinical research on invasive methods in humans, while the animal models used in research and drug discovery possess species-specific differences (Du and Parent, 2015; Grainger et al., 2018). Therefore, brain-on-a-chip models generated with MEAs and human pluripotent stem cell (hPSC) technology are promising tools for resolving these existing challenges (Shafer, 2019; Cecen et al., 2023).

Conventional in vitro epilepsy models, i.e., single neuronal network cultures with standard MEAs, provide information on drug-induced seizure initiation (Odawara et al., 2016; Taga et al., 2019; Alsaqati et al., 2020; Que et al., 2021) but lack the spatial separation to mimic the seizure progression to distal regions that occurs in the brain. The trend in the field to resolve this limitation is to produce brain-on-a-chip models containing compartmentalized microfluidic MEA platforms with structural confinements to achieve the desired network architecture, not only for studying seizures but also for mimicking brain functions more accurately (Taylor et al., 2003; le Feber et al., 2015; DeMarse et al., 2016; Gladkov et al., 2017; Forró et al., 2018; van de Wijdeven et al., 2019; Brofiga et al., 2022). The circular tripartite network concept introduced previously (Pelkonen et al., 2020) provides microfluidic isolation and allows axonal connectivity among three separate neuronal networks, and the embedded MEA enables recording of both local and global electrophysiological activity patterns in the closed circuitry.

To date, most signal analysis algorithms for MEA recordings have been developed for the analysis of single neuronal networks. These algorithms include different approaches based on the interspike interval (ISI; Pasquale et al., 2010; Bakkum et al., 2014; Välkki et al., 2017; Matsuda et al., 2018) and population firing rate (PFR; Wagenaar et al., 2006; Hedrich et al., 2014). These approaches do not fit the analysis of the interconnected compartmentalized networks per se. Previously, network-level synchronous bursting activity and functional connectivity evaluations of compartmentalized MEA data were performed (le Feber et al., 2015; DeMarse et al., 2016; Gladkov et al., 2017; Forró et al., 2018; Brofiga et al., 2022) in the context of different research questions and distinct network designs. Thus, the analysis of neuronal signaling in the circular tripartite network model is suboptimal with existing tools.

In this paper, we performed a joint implementation of custom analysis tools with multiple parameters to evaluate the interrelations within the circular tripartite network model. We developed and applied a local network-level and global circuitry-level burst analyzer. Additionally, as the recorded MEA signals embed extracellular action potentials (APs) and local field potential (LFP) fluctuations (Belitski et al., 2008; Obien et al., 2015), it is beneficial to utilize analysis methods that not only focus on APs but also take alterations in the LFP into account (Trujillo et al., 2019; Pelkonen et al., 2022). Therefore, we adapted an existing functional connectivity assessment tool, the correlated spectral entropy (CorSE) method (Kapucu et al., 2016). With the developed tools, we investigated the synchronization and connectivity alterations induced by kainic acid (KA) exposure which has been used as seizure-like activity-inducing agent in vitro (Odawara et al., 2016; Mzezewa et al., 2022; Zhai et al., 2023). The applied method of local network-level and circuitry-level burst analysis revealed novel in vitro synchronous activity patterns and a variety of effects caused by KA addition into the proximal compartment itself, e.g., the KA-exposed compartment, and the nonexposed distal compartments. In addition, functional connectivity analysis confirmed the formation of networks of various topologies and strengths and revealed noticeable shifts in average connectivity within and between the compartments induced by KA exposure. The circular tripartite network concept combined with the established thorough analysis pipeline creates a promising platform for in vitro research on neurological disorders and for epilepsy research in particular.

## Materials and Methods

### Custom MEA with a microfluidic device

The previously introduced circular tripartite network platform MEMO contains a custom MEA combined with a microfluidic device made of polydimethylsiloxane (PDMS). For device fabrication, a modified version of the micro- and millimeter-scale molding method was used (Ristola et al., 2019). The device (⌀, 22 mm; height, 4 mm) integrates three pentagonal compartments with 50 microtunnels between each pair of compartments. The microtunnels are 3.5 µm in height, 10 μm in width, and 400 μm in length. The edge-to-edge distance between the microtunnels is 35 μm. The device was described previously in more detail (Pelkonen et al., 2020).

The custom MEAs were fabricated in accordance with a previously published process (Ryynänen et al., 2018). In brief, the MEAs comprised 300 nm Ti tracks with an additional 300 nm ion beam-assisted *e*-beam-deposited titanium nitride coating on electrodes organized on a 49 × 49 × 1 mm soda lime glass substrate. For insulation, a 500 nm layer of plasma-enhanced chemical vapor-deposited silicon nitride was used. Each MEA included three areas that matched the compartments in the microfluidic device with 24 circular (⌀, 30 μm) electrodes each. The distances between the electrodes were between 200 and 205.9 μm. A single large reference electrode covered the area from the center of the MEMO through the middle of each compartment. The MEAs were designed to be compatible with an MEA2100 120-electrode headstage (Multi Channel Systems; Pelkonen et al., 2020).

A similar gas supply system for prolonged MEA measurements was used as described in previous publications (Kreutzer et al., 2012; Metsälä et al., 2018). The gas supply chamber was manufactured from polypropylene-like material (RGD430, Object 30, Stratasys) by 3D printing. A sealing PDMS gasket (inner ø, 24 mm) was placed between the MEA plate and the gas supply chamber. The plastic lid (thickness, 100 μm; ⌀, 24 mm) was laser-cut from a polyester sheet. Details of the assembly of the custom MEA, microfluidic device, and additional parts were described previously (Pelkonen et al., 2020).

### Differentiation of cortical neurons and cell culturing on MEMOs

The female human embryonic stem cell (hESC) line Regea 08/017 (Skottman, 2010) was used in this study. The Faculty of Medicine and Health Technology of Tampere University was granted approval from the Finnish Medicines Agency for research involving human embryos (Dnro 1426/32/300/05). A supportive statement was provided by the Regional Ethics Committee of the Expert Responsibility Area of Tampere University Hospital for the derivation, culture, and differentiation of hESCs (R05116). Cortical neuronal differentiation was implemented as described previously (Hyvärinen et al., 2019). Predifferentiated cells were plated on MEMO on Day 32 of maturation, which is referred to as days on MEMO (DOM) 0. A total of 72,500 cells (cell density, 286,000 cells/cm^2^) were plated on each compartment of the platform (Pelkonen et al., 2020). The cells were plated to MEMOs on the same day, and the functional maturation was followed simultaneously in all of them. The timeline of predifferentiation, functional maturation, and KA exposure of the cultures is presented in the Visual Abstract.

### Electrophysiological recordings

Electrophysiological data were recorded with an MEA2100 system (Multi Channel Systems). The temperature of the MEA headstage was held constant at +37°C. To avoid evaporation and contamination during the measurements, the medium chambers were covered with a sterile transparent plastic lid. The raw data were acquired at a sampling rate of 25 kHz. To monitor the development of network activity, standard 10 min recordings were performed twice a week up to DOM 52. For recordings exceeding 10 min, the pH and osmolality of the medium were maintained by covering the chambers with a sterile plastic lid and feeding them with cell culture gas (5% CO_2_, 19% O_2_, and 76% N_2_) using the gas supply chamber (Pelkonen et al., 2020). The recordings with prolonged intervals of excessive spiking spreading simultaneously over most of the recording electrodes were considered noisy and discarded from further analysis (such noisy patterns are easily distinguishable on raster plots as abnormal concentrated activity spots with bin width normalized firing intensities over 40 Hz). The rejection of the data was validated by the examination of the raw signals to confirm the presence of waveform perturbations of presumably nonbiological origin.

### KA exposure and collection of MEA data

The experimental procedure involved inducing seizure-like activity via acute exposure of KA (5 μM, K0250; Sigma-Aldrich; Odawara et al., 2016; Pelkonen et al., 2020; Mzezewa et al., 2022) to the C compartment of the MEMO (hereafter referred to as the proximal compartment). KA was selected because it has been used as seizure-like activity-inducing agent in vitro (Odawara et al., 2016; Mzezewa et al., 2022; Zhai et al., 2023). Pharmacological experiments were performed after the emergence of synchronous bursts spanning over all three compartments which indicated functional maturation of the three compartment networks (DOM 55–62). The assessment data consisted of baseline recordings lasting 30 min, followed by sequential 30 min recordings after KA exposure. The data were subsequently transformed into HDF5 format for further storage and processing. The size of each 30 min recording was ∼6.5 GB. Compartmental information was preserved for the assessment of the exposed compartment C and two nonexposed compartments, A and B (hereafter referred to as distal compartments). Eleven MEMOs from a previously published work (Pelkonen et al., 2020) that demonstrated network activity in all compartments before and after KA exposure were used for tool development and further network-level and novel circuitry-level activity assessment.

### Spike detection

The raw data were processed in MATLAB (MathWorks). Spike detection was performed with the stationary wavelet transform-based Teager energy operator (SWTTEO) algorithm (Lieb et al., 2017), which was revised for biological data (Mayer et al., 2018). The algorithm was implemented in a custom MATLAB data analysis pipeline. The data were prefiltered with an elliptic bandpass filter with a 200 Hz lower passband and a 3,000 Hz upper passband. Initial spike detection was performed with a threshold-based detector with a threshold value of 4.5× the estimate of the noise standard deviation (SD; Quiroga et al., 2004). Next, the SWTTEO algorithm was used to remove putative false-positive spikes. The number of spikes extracted by the threshold-based detector was fed into the SWTTEO algorithm, and the corresponding number of spikes was detected. Only the spikes detected by both methods were accepted as true positives (Hyvärinen et al., 2019). The detected spikes were stored as .csv files, which included the electrode label and time stamp of each detected spike. The spike lists from a previously published work (Pelkonen et al., 2020) were used for network- and circuitry-level burst analysis.

### Building a tool to assess network and circuitry behavior in the MEMO platform

**Figure 1.**
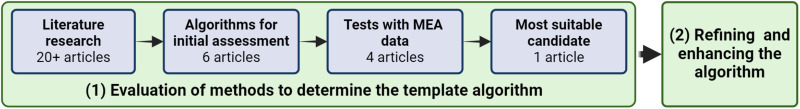
Stages of the development of the method. (1) Pipeline of algorithm evaluation and template selection and (2) iterative feature enhancement process.

A novel tool capable of assessing local network- and global circuitry-level synchronous behavior specifically designed for multicompartmental MEA analysis was developed. We utilized the current knowledge in the field and performed iterative development based on existing synchronous activity detection approaches for MEA data. This process involved (1) an initial evaluation of methods to determine the template algorithm and (2) the refinement and enhancement of the selected algorithm through iterative feature enhancement (Fig. 1).

(1) EVALUATION OF METHODS TO DETERMINE THE TEMPLATE ALGORITHM

To select the most promising approaches for network burst (NB) detection, a comprehensive evaluation of existing techniques was performed. The literature review included >20 publications. The reviewed methods were mainly based on ISI and PFR. Six approaches were preselected for detailed investigation. For the four of them, we managed to obtain the codes from the authors and performed tests to reveal the most suitable candidate. To introduce a broader spectrum of existing methods, all six initially preselected methods are briefly explained below under their corresponding article titles:

- “Network-wide adaptive burst detection depicts neuronal activity with improved accuracy”
  ISI histograms are calculated for each electrode separately and combined into one histogram. Cumulative moving averages are obtained for the bins of the resulting histogram. The maximum value is used to set burst and tail thresholds for detecting bursts in individual electrodes. The synchronization of the single-channel bursts is evaluated by summing across the channels and forming the total NB signal, which is then thresholded according to the minimum participating channels criterion (Välkki et al., 2017).
- “A self-adapting approach for the detection of bursts and NBs in neuronal cultures”
  First, burst detection is performed for each channel separately using the ISI histogram-based method. Then, an interburst interval (IBI) histogram is constructed for the intervals between the starting times of single-channel bursts, and the IBI threshold for combining these bursts into NBs is defined. Only NBs that meet the defined criterion for minimum participating channels are considered (Pasquale et al., 2010).
- “Parameters for burst detection”
  First, the time points of all spikes from the electrodes are combined into a single train to calculate the logarithmic ISI_N_ histogram between *N* consecutive spikes. A histogram exhibiting two separate peaks is assumed to represent two different firing regimes, i.e., spikes that belong to NBs and spikes outside of network firing. Thus, the threshold for detecting NBs is selected as the minimum point between these peaks (Bakkum et al., 2014).
- “An extremely rich repertoire of bursting patterns during the development of cortical cultures”
  The author provided the updated version of the algorithm (https://github.com/wagenadl/octave-wagenaarlab/releases/tag/v0.2.40). The PFR is calculated by summing spikes from all electrodes into 5 ms time bins, after which the data are smoothed. To set the firing rate threshold, the smoothed bins are sorted into the number of bins over firing rate histogram. The exponential function is fit to the area of the histogram to the right of its peak. The first bin in which the real histogram exceeds the fit function defines the firing rate threshold. NBs are defined as contiguous regions in smoothed binned recordings during which the PFR exceeds the threshold (Wagenaar et al., 2006).
- “Detection of synchronized burst firing in cultured human-induced pluripotent stem cell-derived neurons using a four-step method”
  The spikes from all electrodes are combined, and the spikes with an ISI <4 ms are assigned to NBs. NBs containing fewer than 20 spikes are discarded. Neighboring NBs are merged if their IBI is <0 ms. Finally, only the NBs containing >3,000 spikes are defined as valid NBs (Matsuda et al., 2018).
- “Impaired AP initiation in GABAergic interneurons causes hyperexcitable networks in an epileptic mouse model carrying a human NaV 1.1 mutation”
  The recording is divided into 5 ms time bins, and the PFR is calculated and smoothed by a Gaussian kernel with a 100 ms SD. NB candidates are identified as intervals with a PFR exceeding the slowly varying 1 s PFR average. The candidates are accepted if their peak firing rate exceeds three SDs of the recording PFR and 10% of the average of the top five peaks and if at least three electrodes contributed. Finally, the NBs are merged if their IBI is 200 ms or less (Hedrich et al., 2014).

The details of all six initially preselected algorithms are summarized in Table 1.

**Table 1.** Summary information of the six preselected methods

Approach	i	ii	iii	iv	v	vi
Detailed description and usage information	Yes	Yes	Yes	Yes	Yes	Yes
Parametrization and adaptivity	Internal adaptivity is due to the nature of the algorithm	The ISI distribution reflects the data specifics	The ISI_N_ distribution reflects the data specifics	Automatic adaptive firing rate threshold is present.	A likely good fit to the data may be achieved with parameter tuning	Four main parameters are available to adjust the detection
Code obtained from the author	Yes	Yes	Yes	Yes	No	No
Code description	Yes	Yes	Yes	Yes	No	No
Verdict for acquired data	Some tendency to break NBs into shorter components	Good performance but NB borders appeared to be widened; NB merging due to tonic spiking in, e.g., one channel	Sufficient adaptivity for the test data; NB borders assigned relatively well	The method detected some short NBs in addition to evident synchronous patterns	Not available	Not available

Visualizations and a quantitative comparison of the NB detection results for approaches i–iv with available codes are shown in Extended Data Table 1-1.

10.1523/ENEURO.0035-24.2024.t1-1 Table 1-1 The figure demonstrates an example of the detection results of the 4 selected algorithms (I, ii, iii and iv) with available codes. A, The default parameters of the methods were used where present. The raster segments visualize the same segment of baseline data from the C compartment of a MEMO. The green and red vertical lines represent the start and end timings, respectively, of the captured local NBs for each method. Very short NB instances detected within the presented segment were not depicted in the raster plots due to the selected temporal resolution, and the number of such NBs is indicated above each raster plot. B, The histogram shows the distributions of NBs’ durations detected by each of the methods when applied to the MEMO data at baseline condition. Download Table 1-1, TIF file.

The four algorithms (i, ii, iii, and iv) with available codes were assessed using default parameters. Overall, most of our data demonstrated well-developed prolonged periods of local synchronous activity (NBs) with durations of a few seconds according to the raster plots. The efficient detection approach should capture the whole synchronous activity periods without breaking those into smaller instances, but also avoiding overextension of NBs' borders and merging of presumably separate NBs. Visual examples of NB detection by these four algorithms using the default parameters are shown in Extended Data Table 1-1*A*. The histogram in Extended Data Table 1-1*B* displays the distributions of NBs' durations detected by each of the algorithms when applied to our MEMO data at baseline condition. From the histogram, it is possible to infer that Algorithm iii smoothly complies with our above-stated efficient NB detection rationale. Then, rounds of parameter tuning were performed to determine the potential of parameter refinement. The datasets for the evaluation procedure included not only three-compartment MEA data but also data recorded with conventional MEAs (Mzezewa et al., 2022).

Of the four tested approaches, we selected NB detection Algorithm iii published by Bakkum et al., (2014) because it showed good performance and sufficient adaptivity with our data. The approach delivered enough flexibility to show sufficient performance with data of various origins recorded with different MEA setups and thus was chosen as the core for the development of a new robust NB detection tool. The main parameter of the pure method is *N*, which refers to the number of consecutive spikes used for ISI_N_ evaluation. We used *N* = 20 for our MEMO data.

(2) REFINING AND ENHANCING THE ALGORITHM: PARAMETERS TO REFLECT SYNCHRONOUS ACTIVITY

**Figure 2.**
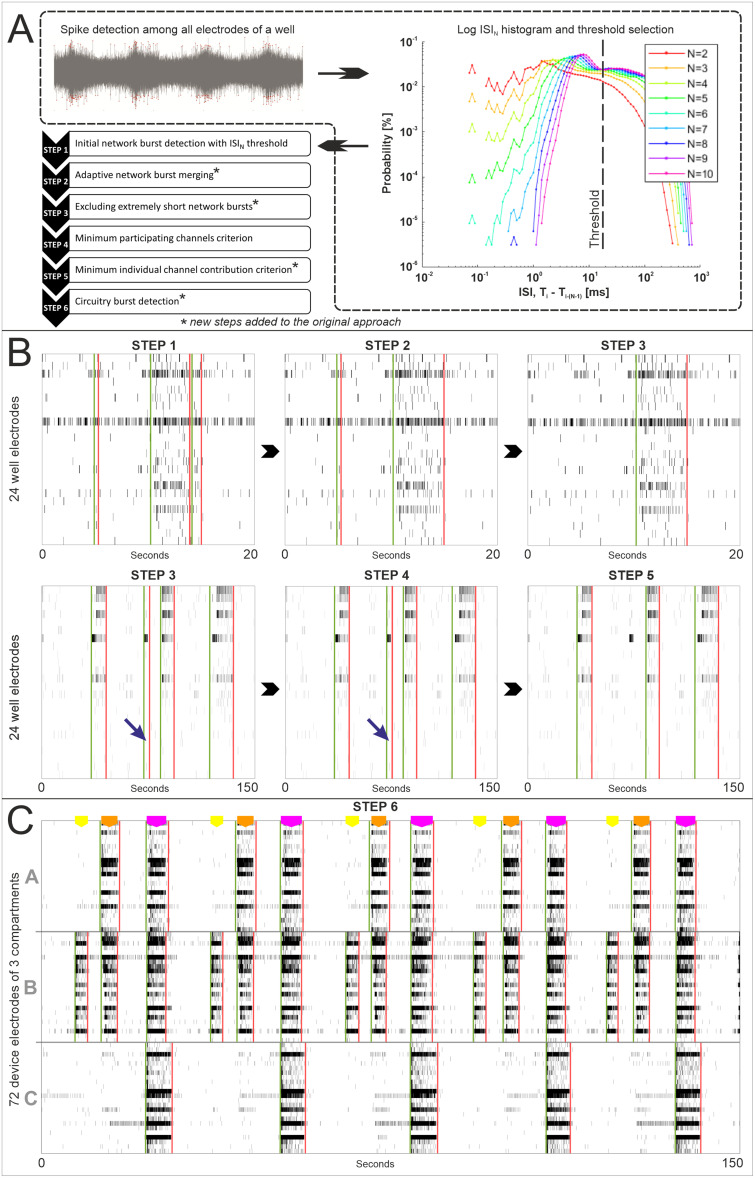
Network and CB detection pipeline. ***A***, Steps of the novel method. The detected spikes are pooled into a histogram, and the ISI_N_ threshold is defined for initial NB detection. ***B***, The first five steps are visualized with single-compartment raster segments of various lengths. The green and red vertical lines represent the start and end times, respectively, of the captured local NBs. The blue arrows in STEPS 3 and 4 indicate limitations of the simple “minimum participating channels” criterion. ***C***, The last step (STEP 6) is shown as a combined raster of three MEMO compartments. The purple polygons at the top depict time intervals with detected circuitry-level bursts involving all three compartments; the orange polygons indicate intermediate circuitry-level bursts with two-compartment synchronicity; and the yellow polygons indicate local NBs in a single compartment. The length of the polygons corresponds to the duration of the corresponding phenomenon.

To achieve an accurate characterization of network activity, various perspectives reflecting different features of the MEA data were evaluated, and a stepwise (STEPS 1–6) feature enhancement process was established (Fig. 2).

- STEP 1: Initial NB detection with the selected algorithm. The selected algorithm was expanded with the following steps to achieve an enhanced and efficient tool for assessing multicompartmental neuronal activity.
- STEPS 2 and 3: Adaptive NB merging and extremely short NB removal.
  To merge instances of short network activity detected on the borders of large NBs, an adaptive merging procedure was developed. The IBI between two NBs was assessed to determine if it was less than the duration of the longest burst in these two NBs multiplied by the merging fraction. If the IBI was lower, then the neighboring NBs were merged. Moreover, an option to remove very short NBs below a duration threshold was introduced. We applied a merging fraction of 0.2 for the data, while a duration threshold was not required for this particular set of electrophysiological recordings.
- STEPS 4 and 5: Minimum participating channels and individual channel contribution criteria. A simple criterion for the minimum number of participating electrodes was set for network activity, as indicated previously in other studies (Pasquale et al., 2010; Bakkum et al., 2014; Välkki et al., 2017). This approach was enhanced by evaluating the level of simultaneous contribution of spatially divergent network locations. A minimum individual channel contribution was defined as a fraction of the mean channel contribution within the detected network activity instance. If the number of spikes detected by the electrode during the NB was below the defined contribution, the electrode was considered to exhibit insufficient participation. The following parameter values were used for processing the data: minimum number of channels needed to constitute an NB, 3/24, and minimum individual channel contribution, 0.15.
- STEP 6: Circuitry burst detection. The detection principle is described in the next subsection below.

### Network- and circuitry-level burst detection and analysis

Separate network analyses of the activity in each of the compartments were used to assess the local networks. STEP 6, the final building block of our new technique, combines local NB detection output to evaluate whole MEMO circuitry-level synchronization. The NBs in the compartments were assessed for temporal alignment to allocate the circuitry-level bursts. In particular, the developed algorithm used the intracompartmental NB times as input and searched for periods where synchronous network activity was detected across the whole MEMO platform. These time segments of full temporal intersection were defined as circuitry-level bursts. The pseudocode for the circuitry-level burst detector is provided as Extended Data.

The analysis involved 11 MEMOs that demonstrated network- and circuitry-level bursts before and after KA exposure in the proximal C compartment. The detection results of the developed method and the contributions of different criteria to the detection results are described in the Results section.

### Test for coincidental alignment of events

To evaluate the possible randomness of intercompartmental synchronization, the resulting data were compared with a simulated number of surrogates (Diggle, 1986; Gladkov et al., 2017). We produced simulated shuffles of temporal alignment of the activities between the compartments. During each simulation, the activity in each compartment of the platform was shifted by a “delta” value randomly chosen within an interval from −20 to 20 s. The cumulative circuitry-level burst duration was used as the test metric that efficiently represented the synchronous alignment of NBs across all three compartments.

If the observed temporal alignment of network events is random, the score of its metric should not deviate substantially from the scores for randomly generated alignments. To assess this, $u_{i}$ was calculated, which determines the differencebetween the metric score $x_{i}$ for alignment i and the average metric scores of all the other alignments:

$$u_{i}=\mathrm{abs}\left(x_{i}-\frac{1}{n-1}\underset{\;j\neq i}{\sum }\;x_{j}\right),$$

where n is the total number of simulations plus 1 observed instance. If the observed alignment is random, $u_{\mathrm{observed}}$ should have a similar magnitude as the u values of the simulated alignments. To evaluate this, an empirical *p* value was calculated by sorting all u values in descending order and dividing the index of $u_{\mathrm{observed}}$ by n. The observed alignment does not fit the random simulations when its *p* ≤ 0.05 at the 95% confidence level.

### Functional connectivity analysis

Functional connectivity analysis was performed to reveal the impact of KA exposure on local network- and circuitry-level connectivity. To assess intra- and intercompartmental connectivity, the CorSE approach was used as described previously (Kapucu et al., 2016). This method evaluates the connectivity strength of two electrode signals based on the correlation of the temporal changes in their spectral contents. Briefly, recordings from each electrode were cut into time windows, and Shannon's entropy of their power spectrum was calculated. The degree of common temporal changes for different electrode pairs was subsequently assessed by calculating the corresponding cross-correlations and obtaining CorSE values. This approach uses raw signal waveforms as input data, and the LFP components of the data contribute to the results. The functional connectivity analysis included all possible pairs of electrodes within and between the three compartments of the MEMO platform. The analysis was performed by implementing a MATLAB code, which was based on a publicly available analysis library (https://www.mathworks.com/matlabcentral/fileexchange/59626-spectral-entropy-based-neuronal-network-synchronization-analysis-corse). The modifications included the introduction of a new electrode layout for data processing and result visualization.

### Code accessibility

The manuscript includes a detailed description of the analysis pipeline. The original code for CorSE-based functional connectivity analysis (Kapucu et al., 2016) can be found in the corresponding repository (https://www.mathworks.com/matlabcentral/fileexchange/59626-spectral-entropy-based-neuronal-network-synchronization-analysis-corse). The code for ISI_N_ threshold-based burst detector can be obtained from the supplementary data of the original publication (Bakkum et al., 2014). The elaborated pseudocode for the circuitry-level burst detector is provided as Extended Data.

10.1523/ENEURO.0035-24.2024.d1 Pseudocode Download Pseudocode, DOCX file.

## Results

### The developed analysis algorithm provides robust identification of network- and circuitry-level bursts

The application of the developed network and circuitry burst (CB) detection tool is visualized in Figure 2. After the initial detection, putative NBs were labeled (STEP 1), and adaptive NB merging was performed (STEP 2). The removal of extremely short NBs (STEP 3) was not used for the MEMO data in this study. A simple criterion of a minimum of three participating channels (STEP 4) was used to confirm network activity. However, when only one channel contributes sufficiently, and the activity of the other channels is truly weak, short NBs are not removed (Fig. 2*B*). Thus, to emphasize the sufficient contribution of the participating channels, the minimum individual channel contribution criterion was applied (STEP 5). It removes detection cases in which an insufficient number of channels actively participate in an NB, while others fire only a few spikes within the duration of the synchronous event. Finally, the algorithm searched for temporally intersecting segments of NBs in the compartments and classified these as circuitry-level bursts (STEP 6; Fig. 2*C*).

The developed tool was used to perform network- and circuitry-level burst detection for MEMO data during baseline and KA exposure conditions. Eight output parameters were derived to reflect various features of the detected activity types, including the mean IBI, mean burst duration, mean spike frequency in bursts, percentage of spikes in bursts, mean spikes in bursts, total number of bursts, mean channels in bursts, and mean dominating channels in bursts (dominating channels are defined as the electrodes that meet the minimum individual channel contribution criterion in STEP 5). Wilcoxon matched-pairs signed-rank test was used to perform pairwise comparisons of each output parameter before and after KA exposure over 11 MEMOs. The comparisons were performed for both local network- and global circuitry-level bursts.

### Randomness assumption rejection: detected circuitry-level bursts are not caused by random alignment of NBs

**Figure 3.**
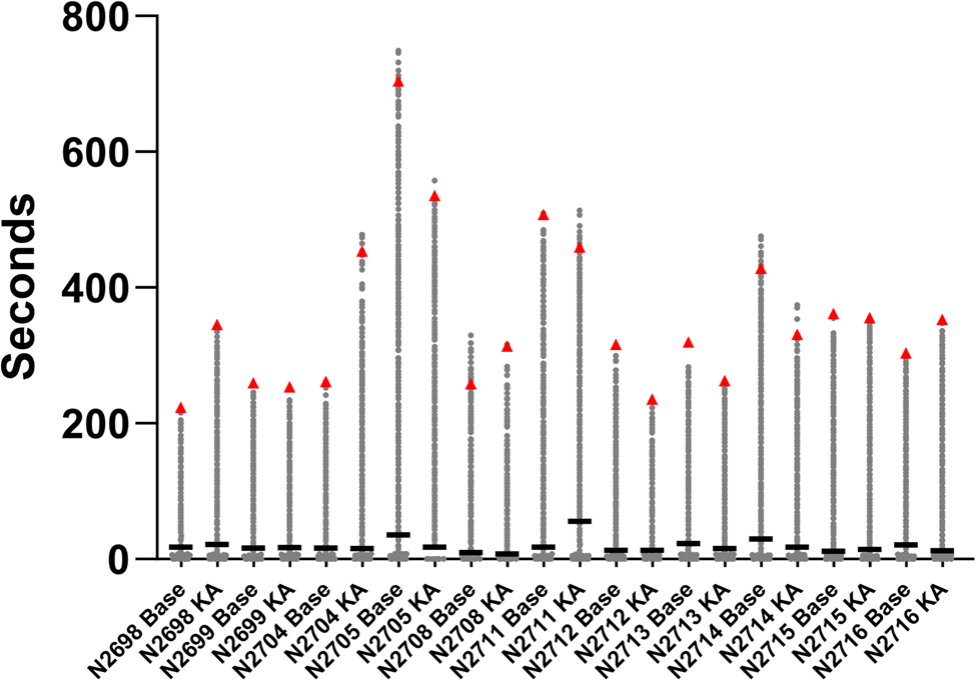
Cumulative circuitry-level burst duration. The scatter plots depict scores for the derived metric among 5,000 simulations for 11 MEMOs labeled with their MEMO platform identification codes. The simulations included compartmental NB shuffling for each plate for two conditions: baseline and KA exposure. The real observed condition scores are indicated with red triangles. The bold line on each scatter plot represents the mean value for the scores of 5,000 simulations together with the single entity calculated from the observed data.

A randomness check was implemented to test whether the alignment of NBs over the three compartments of MEMO was coincidental. The cumulative duration of three-compartment circuitry-level bursts was the test metric, and its observed score was compared with the scores obtained from 5,000 surrogate simulations using the calculated u values. The results indicated that the u values of the surrogate data were significantly different from the $u_{\mathrm{observed}}$ values of the original data (Fig. 3). For the cumulative circuitry-level burst duration, all the empirical *p* values derived using the u values were <0.005. Thus, the randomness assumption was rejected.

### Characterization of synchronous activity patterns in circular tripartite networks

Nonrandom alignments of the synchronous activity patterns in the circular tripartite networks were encountered and characterized at three levels. The level of synchronization within a single network in a separate compartment was considered a local NB. The synchronous NB activity that spread across the whole circular tripartite network in three compartments was considered a CB. Additionally, an intermediate synchronous activity pattern was encountered with two compartments orchestrating simultaneously, which was defined as an intermediate circuitry burst (ICB). Our analysis showed that a single network can fire NBs and participate in both ICBs and CBs. Figure 2*C* depicts the detected NBs, ICBs, and CBs as yellow, orange, and purple polygons, respectively, on the top of the raster.

We performed a baseline variability assessment of the newly characterized synchronous activity patterns. The numbers of patterns detected for 11 MEMOs at baseline are shown in Table 2.

**Table 2.** Numbers of synchronous patterns detected for 11 MEMOS at baseline

	N2698	N2699	N2704	N2705^a^^,^^b^	N2708^a^^,^^b^	N2711^a^^,^^b^	N2712^b^	N2713	N2714^a^^,^^b^	N2715^b^	N2716
NB A	158	189	126	179	70	121	68	85	113	76	74
NB B	85	64	189	179	70	121	130	161	113	76	74
NB C	152	128	63	179	70	121	68	168	113	159	84
ICB AB	10	0	64	0	0	0	0	2	0	0	13
ICB BC	0	0	0	0	0	0	0	82	0	0	0
ICB AC	76	64	0	0	0	0	0	0	0	0	0
ICB total	86	64	64	0	0	0	0	84	0	0	13
CB	76	64	63	179	70	121	68	85	113	76	61

The top row shows the MEMO platform identification code. The table lists the numbers of detected synchronous activity patterns among 11 MEMOs at baseline. ICB labels are created from the two letters of the corresponding involved compartment labels. The ICBs that were associated with the CBs were not considered and hence were not included in the table. ICB refers to intermediate circuitry burst, and CB refers to circuitry burst. The ^a^ symbol indicates the cases of full synchrony in the network, and the ^b^ symbol indicates the cases without intermediate circuitry synchronization. Table 2-1 presents the numbers of the different synchronous patterns after KA exposure.

10.1523/ENEURO.0035-24.2024.t2-1 Table 2-1 The top row shows the MEMO platform identification code. The table lists the numbers of detected synchronous activity patterns among 11 MEMOs after KA exposure. ICB labels are created from the two letters of the corresponding involved compartment labels. The ICBs that were associated with the CBs were not considered and hence were not included in the table. ICB refers to intermediate circuitry burst, and CB refers to circuitry burst. Download Table 2-1, DOCX file.

All MEMOs expressed CBs in varying numbers, from 61 to 179, during 30 min recordings. The analysis results showed that four MEMOs (36%) exhibited full synchrony, where all the local network synchronous activity was a part of the circuitry-level bursts (indicated with the ^a^ symbol in Table 2). Moreover, six MEMOs (55%) exhibited CB-level synchronous patterns without any additional ICBs (indicated with the ^b^ symbol in Table 2). Among the remaining MEMOs (5/11, 45%), CBs were accompanied by ICBs, of which the number and involved compartments varied.

In conclusion, with the developed analysis tool, it was possible to reveal the circuitry-level organization of synchronous activity in circular tripartite networks and successfully extract novel features: CBs and ICBs.

### The developed algorithm revealed KA-induced changes in activity at different synchronization levels

Following the baseline variability assessment, we evaluated the KA-induced changes in the numbers of detected synchronous activity patterns. The numbers of different synchronous patterns after KA exposure are provided in Extended Data Table 2-1. The MEMOs showed gradual changes in activity, especially in the proximal C compartments. All four cases of full-circuit synchronization discussed above and marked with ^a^ in Table 2 were disturbed, and four of the six cases with no ICBs at baseline started to exhibit ICBs. In general, the CB and total ICB numbers demonstrated mixed dynamics among the plates after exposure. The number of CBs increased for seven MEMOs (64%) and decreased for four MEMOs (36%). The total number of ICBs increased for five plates (46%), decreased for three plates (27%), and remained the same for three plates (27%).

We subsequently studied the effect of KA exposure on NB and CB characteristics. We conducted pairwise evaluations of the results by comparing the baseline output parameters with their values after KA exposure for each MEMO. This approach allowed us to investigate the overall effects of KA exposure on our newly defined synchronous activity patterns. Additionally, we studied the time-variant impacts of KA exposure as percent changes to enhance the representativeness of the data, taking into account the inherent baseline variability.

**Figure 4.**
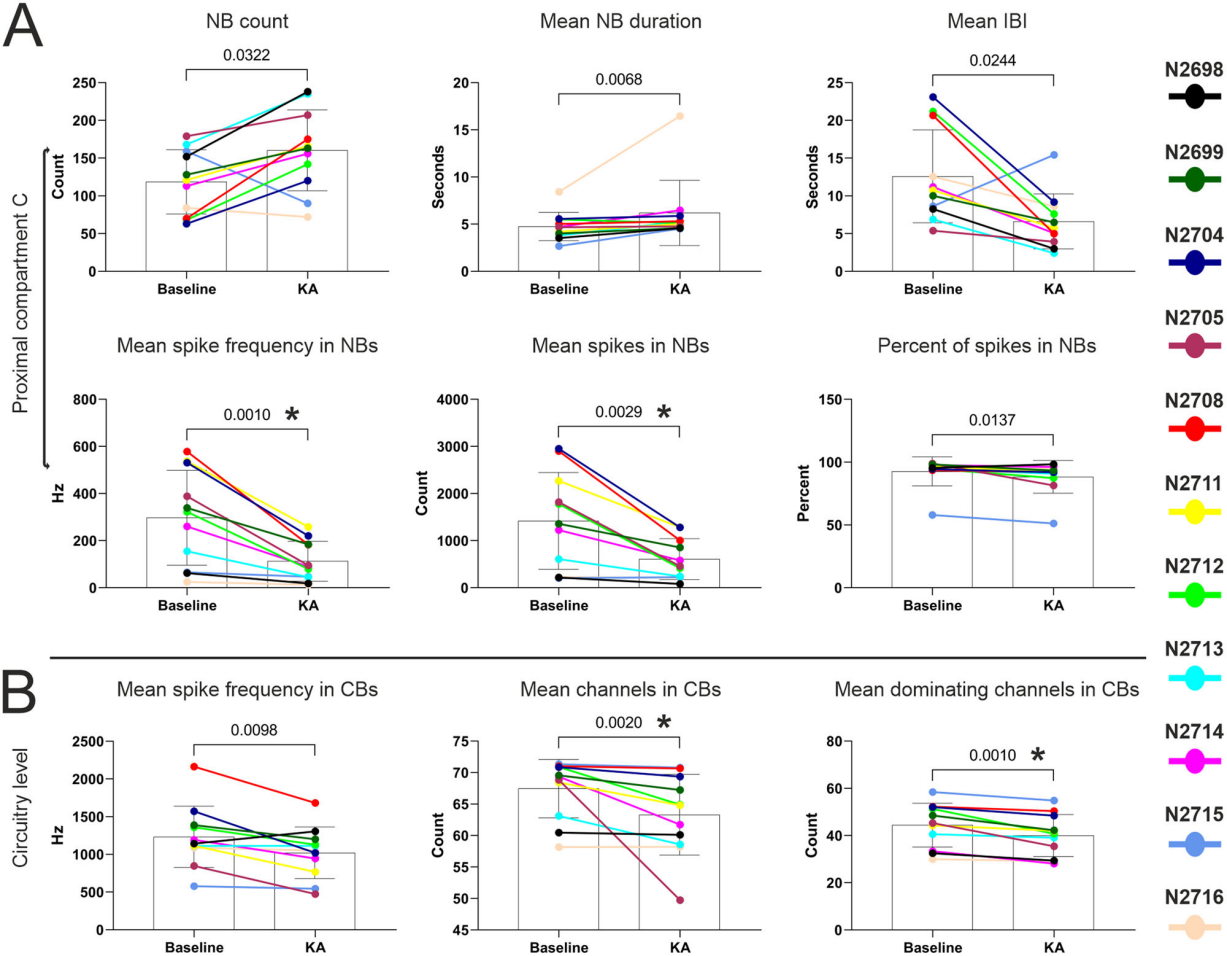
Pairwise comparisons. ***A***, Six of the eight NB output parameters from the proximal C compartment and ***B***, Three of the eight CB output parameters that showed significant (*p* < 0.05) KA-induced alterations prior to Bonferroni’s adjustment of the significance level. Wilcoxon matched-pairs signed-rank test. The color codes for the corresponding MEMO labels are shown on the side. The bar charts with whiskers present means and standard deviations. *n* = 11 for each pairwise comparison. The *p* values are presented on top of each plot. The NB/CB parameters with alterations that remained significant (*p *< 0.00625) after Bonferroni’s correction for the families of eight hypotheses are marked with asterisk *. The full lists of pairwise comparisons for the NB and CB parameters are available in Extended Data Figures 4-1–4-4.

The KA-exposed proximal C compartments of the MEMOs exhibited prominent changes in the output parameters of NBs. Figure 4*A* shows pairwise comparisons of six out of the total eight NB output parameters for the proximal compartment. Prior to any correction for a multiple-comparisons case, the significant (*p* < 0.05) increases in NB number and mean NB duration and decreases in mean IBI, mean spike frequency in NB, mean spikes in NB, and percentage of spikes in NB were observed. However, since multiple Wilcoxon tests were performed for the parameters derived from the same MEA activity set, it is reasonable to consider the correction. Bonferroni’s adjusted significance level of α = 0.05 for the family of eight hypotheses equals 0.00625. Thus, the decreases of mean spike frequency in NB and mean spikes in NB in the C compartment remained significant (*p* < 0.00625) after Bonferroni’s correction. On the other hand, significant (*p* < 0.05) KA-induced decreases of three out of the total eight output parameters were observed at the CB level prior to Bonferroni’s correction: of the mean spikes in the CB, mean channels in the CB, and mean dominating channels in the CB (Fig. 4*B*). After the correction for multiple comparisons of the total eight circuitry-level parameters, the decreases in mean channels in the CB and mean dominating channels in the CB remained significant (*p* < 0.00625). Only the NB/CB parameters that showed significant alterations after Bonferroni’s correction are marked with * in Figure 4. In conclusion, newly defined CB parameters revealed the impacts of KA exposure at the global circuitry level in addition to the changes observed in the proximal compartment where KA was applied. The full lists of pairwise comparisons are available in Extended Data Figures 4-1–4-4.

10.1523/ENEURO.0035-24.2024.f4-1 Figure 4-1 Pairwise comparisons of NB parameters before and after KA treatment in the distal A compartment. The color codes for the corresponding MEMO codes are shown on the side. The bar charts with whiskers present means and standard deviations. n = 11 for each pairwise comparison. *p* values are presented on top of each plot. The Wilcoxon matched-pairs signed-rank test was used. Download Figure 4-1, TIF file.

10.1523/ENEURO.0035-24.2024.f4-2 Figure 4-2 Pairwise comparisons of NB parameters before and after KA treatment in the distal B compartment. The color codes for the corresponding MEMO codes are shown on the side. The bar charts with whiskers present means and standard deviations. n = 11 for each pairwise comparison. *p* values are presented on top of each plot. The Wilcoxon matched-pairs signed-rank test was used. Download Figure 4-2, TIF file.

10.1523/ENEURO.0035-24.2024.f4-3 Figure 4-3 Pairwise comparisons of NB parameters before and after KA treatment in the proximal C compartment. The color codes for the corresponding MEMO codes are shown on the side. The bar charts with whiskers present means and standard deviations. n = 11 for each pairwise comparison. *p* values are presented on top of each plot. The Wilcoxon matched-pairs signed-rank test was used. Download Figure 4-3, TIF file.

10.1523/ENEURO.0035-24.2024.f4-4 Figure 4-4 Pairwise comparisons of CB parameters before and after KA treatment at the circuit level. The color codes for the corresponding MEMO codes are shown on the side. The bar charts with whiskers present means and standard deviations. n = 11 for each pairwise comparison. *p* values are presented on top of each plot. The Wilcoxon matched-pairs signed-rank test was used. Download Figure 4-4, TIF file.

**Figure 5.**
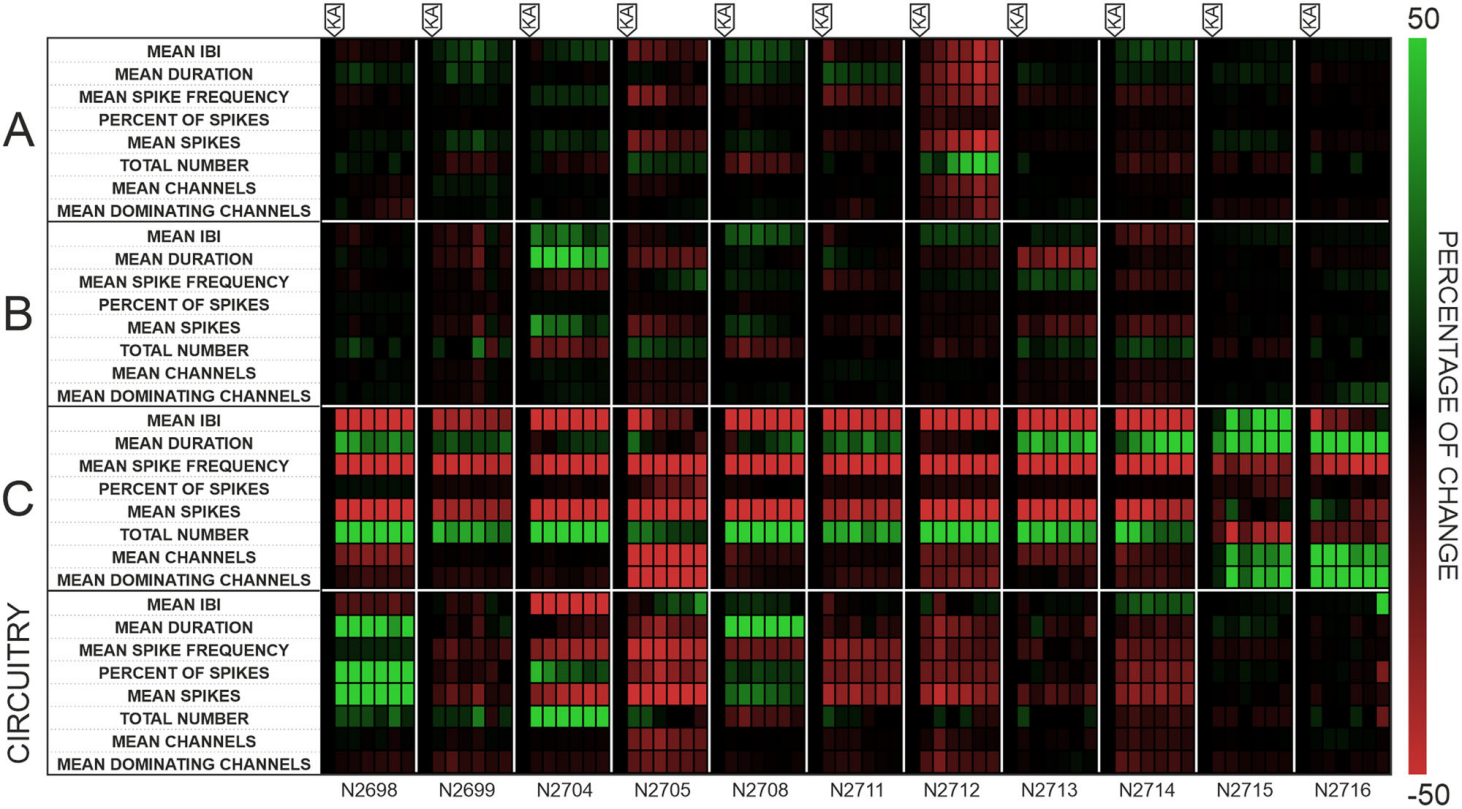
Binned heatmap of baseline-normalized percentage changes in eight parameters in three compartments and at the circuitry level among 11 MEMOs. The last 5fmin bin of the baseline recording was used to calculate the percentage change in the subsequent six 5fmin bins recorded after KA exposure. The upper and lower limits of percentage change were set to 50% for visualization purposes.

In addition, the output parameters were followed after KA exposure to study the time-variant impacts. These impacts are visualized on a binned heatmap with a bin width of 5 min in Figure 5. Each parameter was normalized to its value during the last 5 min baseline recording, which is plotted in black. Increasing and decreasing changes are indicated by red and green, respectively, where changes up to 50% are indicated with gradients. The binning results for the proximal C compartment confirmed that KA exposure induced a steady increase in the NB number and resulted in a slight increase in the NB duration (except for two MEMOs, N2705 and N2712), although the latter parameter showed some variability over time among the MEMOs. On the other hand, the spike frequency in NBs (all MEMOs), mean spikes in NBs (all MEMOs except for N2715), and IBIs (all MEMOs except for N2715) showed stable downtrends in the proximal compartment. For the mean spike frequency and mean spikes at the CB level, downtrends were also partly observed. In the distal compartments, most NB parameters demonstrated mixed responses of relatively low intensity with more temporal variability (Fig. 5).

These results support the initial observations depicted in Figure 4. In conclusion, the time-binning analysis of the studied parameters revealed a relatively robust response in the proximal C compartment and partly at the CB level. The magnitude of percentage changes, and trends were more evident in the proximal compartment and at the CB level than in the distal compartments.

### Functional connectivity analysis

Functional connectivity analysis was performed via CorSE values to explore the effects of KA exposure on local and circuitry-wide network connectivity dynamics. This analysis provided a comprehensive representation of changes after KA exposure by including its impacts on LFPs in addition to investigating CB parameters based on spike alignment.

**Figure 6.**
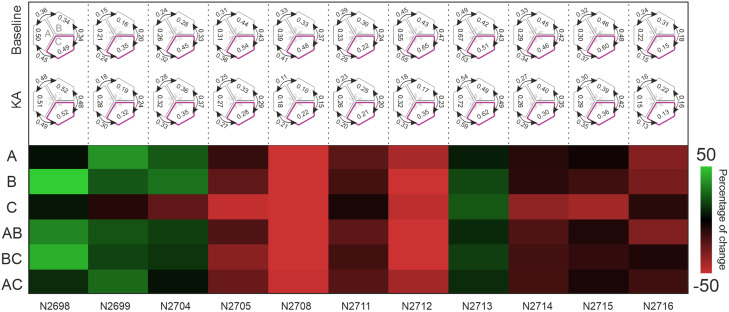
Functional connectivity analysis results before and after KA exposure. In the upper half of the figure, the average intracompartmental and intercompartmental average connectivity values are plotted for both experimental conditions for all 11 MEMOs. The exposed proximal C compartment is highlighted in purple. The first scheme embeds the compartment labels. The lower half of the figure shows the heatmap of percentage changes in the connectivity values after KA exposure. The upper and lower limits of percentage change were set to 50% for visualization purposes. Extended Data Figure 6-1 shows pairwise comparisons of average intra- and intercompartmental CorSE values before and after KA exposure among 11 MEMOs. Extended Data Figure 6-2 presents pairwise comparisons of average intra- and intercompartmental CorSE values within each MEMO.

Before KA exposure, there was noticeable variability in the initial baseline average intra- and intercompartmental CorSE values among the MEMOs, indicating diverse connectivity patterns (Fig. 6). Changes in intra- and intercompartmental CorSE values after KA exposure highlighted specific alterations in functional connectivity induced by KA (Fig. 6). Remarkably, KA exposure led to a significant decrease in average connectivity values in 9 out of 11 MEMOs (82%) within the proximal C compartment, while two MEMOs (N2713 and N2698, 18%) exhibited increased connectivity values (Extended Data Fig. 6-1). Furthermore, most MEMOs showed a consistent response pattern throughout the network: intra- and intercompartmental connectivity mostly followed the direction of change observed in the proximal compartment (except for N2699 and N2704; Extended Data Fig. 6-2). This finding implied that KA had a global impact on network communication and shed light on the vulnerability or resilience of the proximal and distal compartments to KA exposure. This was evident from the significantly altered local NB parameters (Fig. 4) and the significant changes in intranetwork connectivity in the proximal compartment (Extended Data Fig. 6-1). On the other hand, the significant changes in CB parameter values and the changes in intercompartmental connectivity imply the broader influence of KA exposure on distal networks.

10.1523/ENEURO.0035-24.2024.f6-1 Figure 6-1 Pairwise comparisons of average intra- and intercompartmental CorSE values before and after KA treatment among MEMOs. The color codes for the corresponding MEMO codes are shown on the side. The bar charts with whiskers present means and standard deviations. n = 11 for each pairwise comparison. *p* values are presented on top of each plot. The Wilcoxon matched-pairs signed-rank test was used. Download Figure 6-1, TIF file.

10.1523/ENEURO.0035-24.2024.f6-2 Figure 6-2 Pairwise comparisons of average intra- and intercompartmental CorSE values within each MEMO. Symbolic codes for corresponding connectivity are shown on the side. Download Figure 6-2, TIF file.

## Discussion

### Circular tripartite network

In this work, we developed analysis tools to assess functional neuronal network interactions in a three-compartment circuitry platform. We utilized MEA data obtained from a previously published model (Pelkonen et al., 2020) to replicate the functional connectivity and communication dynamics of interconnected networks. The novel analysis tools identified different levels of bursting activities, including novel circuitry-level bursting behavior not previously reported for in vitro neuronal networks. Additionally, we assessed intra- and intercompartmental connectivity strengths and, most importantly, revealed alterations in these features after KA exposure. We demonstrated KA-induced functional alterations in the exposed network and confirmed that some of these alterations propagated to distal regions in the circuitry. Moreover, we revealed disturbances that also reflected the global functionality of the whole circuitry system. Furthermore, our results strongly support the conclusions about the successful development of functional, interconnected three-compartment cultures (Pelkonen et al., 2020) and provide an effective means of quantitatively assessing the acquired data.

### Investigating neuronal activity in multicompartmental MEA data

To fully leverage the advantages of a three-compartment platform, suitable analysis tools for the obtained electrophysiological data must be designed. Analyzing network synchrony is crucial for obtaining a deeper understanding of epileptic seizure-like activity in vitro since it is suggested that neuronal network synchrony is a key factor in the onset and spread of seizures (Jiruska et al., 2013; Ren et al., 2021; Mulcahey et al., 2022). In the context of the tripartite network circuitry model, by analyzing the synchronous patterns of firing within and between the interconnected local networks in vitro, one can identify interrelations of induced hyperactivity that may contribute to understanding the initiation and spread of epileptic seizures in vivo. For example, the MEMO platform may provide a means for modeling local abnormal activity spread from the seizure-onset zone to distal cortical regions, which evokes global network activity changes (Piper et al., 2022). Hence, our goal is not only to thoroughly evaluate the synchronous bursting of local networks in compartments but also to determine the characteristics of global circuitry synchronization to reveal the effects of single-compartment KA exposure.

Several works on multicompartmental MEA data analysis included synchronous bursting features (le Feber et al., 2015; DeMarse et al., 2016; Gladkov et al., 2017; Forró et al., 2018; Brofiga et al., 2022). In relation to our study protocol, experimental designs with multiple electrodes in each compartment were considered of greatest interest. Most works followed the same general method: first, NBs were detected inside the compartments separately using traditional approaches, and then, the detected NB outcomes were evaluated for temporal alignment (le Feber et al., 2015; DeMarse et al., 2016; Gladkov et al., 2017). We decided to follow a similar approach and created an efficient algorithm for intracompartmental NB detection and extrapolated the results to the circuitry level. In addition to synchronous bursting analysis, several correlation-based techniques have been implemented to verify functional connectivity and signal propagation between the compartments of multicompartmental MEAs (le Feber et al., 2015; DeMarse et al., 2016; Kapucu et al., 2016; van de Wijdeven et al., 2019; Brofiga et al., 2022). We chose to use the correlation spectral entropy approach (Kapucu et al., 2016), which takes the raw MEA signal as input data and evaluates the functional connectivity between electrode pairs based on the whole spectrum of the recorded signal. Thus, the fundamental advantage of the method is its independence from any preceding feature extraction procedures such as spike detection.

In summary, we used a multilevel synchronous bursting analysis tool supported by the functional connectivity assessment method. Thus, we demonstrated the circular tripartite network formation and established a comprehensive analysis pipeline, providing additional information on the different patterns and levels of synchronization in interconnected neuronal networks.

### Development of a network and CB detector for novel MEA-embedded platforms

Since the existing analysis tools for conventional MEA platforms (Wagenaar et al., 2006; Bakkum et al., 2014; Hedrich et al., 2014; Välkki et al., 2017; Matsuda et al., 2018) are not suitable for multilevel burst analysis, we extended the general idea of synchronous network activity analysis. The approach was selected, and custom algorithm development was performed based on the existing algorithms, which are not meaningful without adaptation or redefinition. Through the iterative development approach, we achieved the desired tool with a multilevel bursting assessment feature. The ISI_N_ threshold-based burst detector (Bakkum et al., 2014) chosen as the local NB detection core provides efficient identification of synchronous network-wide activity. Unlike in the original study, relatively large bursting events rather than synchronous activity instances several milliseconds long were captured. Thus, refinement and additional features were utilized in the developed algorithm. Previously, burst-related spiking activity was defined as prebursts and burst tails (Pasquale et al., 2010; Kapucu et al., 2012); hence, merging close bursting activities has been suggested to correct erroneous detections (Kapucu et al., 2012; Hedrich et al., 2014; Matsuda et al., 2018). Our additional merging step was designed to inherit adaptivity to the length of adjacent NBs to avoid using fixed values lacking universality. Additionally, extremely short NBs, mostly considered false detections, tend to affect the analysis output. Although this issue was not evident in the analyzed dataset, we added a short NB removal option to extend the functionality of our tool, as suggested previously (Wagenaar et al., 2006). As true NBs should incorporate spikes from several electrodes in a compartment, we added an extension of the basic minimum participating channels criterion by checking individual channel contributions.

The final analysis step processes NB detection outcomes in each individual compartment to capture possible CB instances: the designed algorithm checks local NB instances for temporal alignment and assigns a CB in the case of three-compartment synchronous NBs. With the high number of microtunnels between the compartments, a minimal delay between the NBs was assumed, as suggested previously (DeMarse et al., 2016); hence, only the time intervals with full three-compartment temporal intersections of NBs were considered CBs. In addition, the nonrandomness of the observed baseline temporal alignment of NBs was tested analogously to the methods of the previous work (Gladkov et al., 2017), which validated the genuine synchronous CB firing of the three-compartment networks. We also performed ICB detection, where the requirement for temporal alignment of local NBs was reduced to two compartments. In summary, the developed detector was appropriate for use with novel data. The revealed synchronous activity patterns were accurately detected, and the detection performance was validated visually by experienced biologists. Thus, three levels of synchronous bursting, NBs, ICBs and CBs, were defined.

Brain network modeling and in vivo research suggest that brain circuits exhibit a nodular organization with established intranetwork and internetwork connectivity (Meunier et al., 2010; Feldt et al., 2011; Bassett and Sporns, 2017; Schröter et al., 2017; Sadaghiani and Wirsich, 2020). The circular alignment of neuronal networks in vitro more closely resembles the connectivity of in vivo networks and potentially allows assessment of more complex activity features (Pelkonen et al., 2020; Duru et al., 2022; Ihle et al., 2022). As synchronous bursting implies the activity of functionally connected brain areas (Kamioka et al., 1996), we suggest that the detected CBs are an appropriate model of synchronous activity of three interconnected cortical regions, while ICBs depict two cortical regions orchestrating together. In this context, local NBs in one compartment represent synchronous activity of a distinct brain node, and KA exposure makes this node model analogous to the seizure-onset zone (Piper et al., 2022).

### KA-induced activity alterations

The most prominent activity shifts were observed in the proximal C compartment exposed to KA; NBs appeared more frequently, but a general shift toward tonic spiking and some switches and alterations in firing intensity among channels were observed. The introduced analysis tool detected quantitative changes in synchronous activity patterns. In the proximal C compartment, there was a prevalent decrease in the mean IBI and an increase in the total number of NBs, while the NB duration increased in some cases. This difference may be caused in part by the elevation of tonic spiking after KA exposure, which affects detection and broadens NBs. As the effect of KA exposure on network bursting has been shown to be concentration dependent, these alterations are consistent with previous results (Zhai et al., 2023). On the other hand, the decreases in the mean spikes in NBs (Odawara et al., 2016) and mean spike frequency in NBs observed in C compartments may indicate that initially condensed synchronous activity breaks down into less prominent but more temporally spread patterns. A slight decrease in the percentage of spikes in NBs supports the idea that activity spreads into more tonic patterns.

The KA-induced activity alterations spread from the proximal compartment to the distal compartments. Similarly, the spread of synchronous activity alterations through distinct cortical regions has been reported during KA exposure in the rat occipital cortex (Jimbo and Robinson, 2000) and hippocampal slices (Fisher and Alger, 1984). However, the strength of the effect of KA on NB output parameters in the nonexposed compartments varied and was generally less evident than that in the C compartments. The weaker response may be a result of resistance to excitation with a proportional increase in inhibition typical of cortical structures (Nelson and Turrigiano, 1998; Shu et al., 2003; Trevelyan and Schevon, 2013); hence, as the networks in nontreated distal compartments receive putative excitation only through synaptic input from the exposed network, they might manage to suppress this excitation. The results obtained with the novel analysis tool were strongly supported by functional connectivity analysis, as the baseline average intra- and intercompartmental CorSE values varied noticeably among the MEMOs, and KA exposure affected these values dissimilarly.

Finally, we present an evaluation of global synchronous activity shifts for circular tripartite networks. The prominent alterations of CB output parameters confirmed the global network effect of KA exposure. We observed significant decreases of mean channels in the CB and mean dominating channels in the CB among the MEMOs even after applying the conservative Bonferroni’s correction. These results indicate the shift in global network orchestration patterns. For example, previous ex vivo studies of brain slices have demonstrated drastic alterations in well-established circuitries as a result of KA exposure (Reid et al., 2008). The functional connectivity analysis provided additional support for thorough effects of KA exposure: average intra- and intercompartmental CorSE values had clear trends within most MEMOs. These results allow us to conclude that exposure noticeably affects the activity of whole circular tripartite networks.

## Conclusions

The functional properties of the circular tripartite networks were thoroughly studied with the developed signal analysis tools. The analysis revealed three levels of synchronous bursting: NBs at the local intracompartment level, ICBs as an interplay of two compartments, and fully matured CBs with synchronous orchestration of all three local networks. KA exposure in the model drastically altered the synchronous activity patterns, especially in the exposed proximal compartment. To some extent, these alterations spread to distal compartments, affecting the global functioning of the circuitry. The circular tripartite network model with advanced analysis is highly relevant for seizure-like activity modeling in vitro. Moreover, it enables the evaluation of spatial propagation of hyperactivity, which is not currently possible with single-network models. Furthermore, the platform with an integrated analysis pipeline has great potential for modeling disease progression in patients with neurodegenerative disorders.

## Synthesis

Reviewing Editor: Jennifer Dulin, Texas A&M University Decisions are customarily a result of the Reviewing Editor and the peer reviewers coming together and discussing their recommendations until a consensus is reached. When revisions are invited, a fact-based synthesis statement explaining their decision and outlining what is needed to prepare a revision will be listed below. The following reviewer(s) agreed to reveal their identity: NONE.

Synthesis statement:

I sincerely apologize for the delay in returning a review of this manuscript to you. There were difficulties in securing additional reviews by the previous reviewers, and for that reason I (the editor) chose to review the resubmission thoroughly, rather than send it out for all new reviews. It is my opinion that all of the previous reviewers' concerns were satisfactorily addressed and that no further changes are required.
