# Supplementary figures and images for: Exploring Kainic Acid-Induced Alterations in Circular Tripartite Networks with Advanced Analysis Tools
Source: eNeuro. 2024 Jul 26;11(7):ENEURO.0035-24.2024. doi: 10.1523/ENEURO.0035-24.2024 (PMC11289587; doi:10.1523/ENEURO.0035-24.2024)

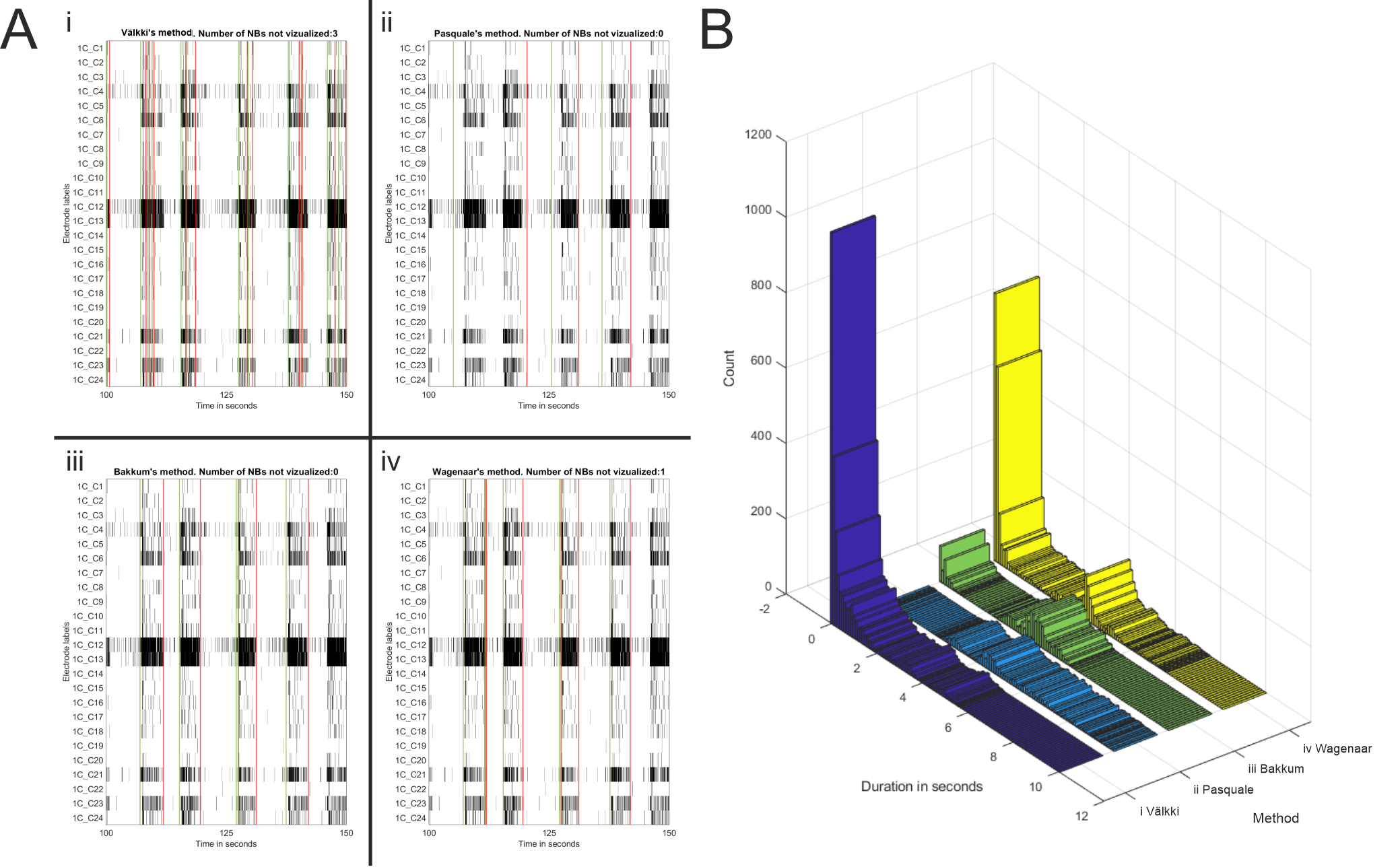

Supplement: Table 1-1 — The figure demonstrates an example of the detection results of the 4 selected algorithms (I, ii, iii and iv) with available codes. A, The default parameters of the methods were used where present. The raster segments visualize the same segment of baseline data from the C compartment of a MEMO. The green and red vertical lines represent the start and end timings, respectively, of the captured local NBs for each method. Very short NB instances detected within the presented segment were not depicted in the raster plots due to the selected temporal resolution, and the number of such NBs is indicated above each raster plot. B, The histogram shows the distributions of NBs’ durations detected by each of the methods when applied to the MEMO data at baseline condition. Download Table 1-1, TIF file. [file eneuro-11-ENEURO.0035-24.2024-s011.tif]

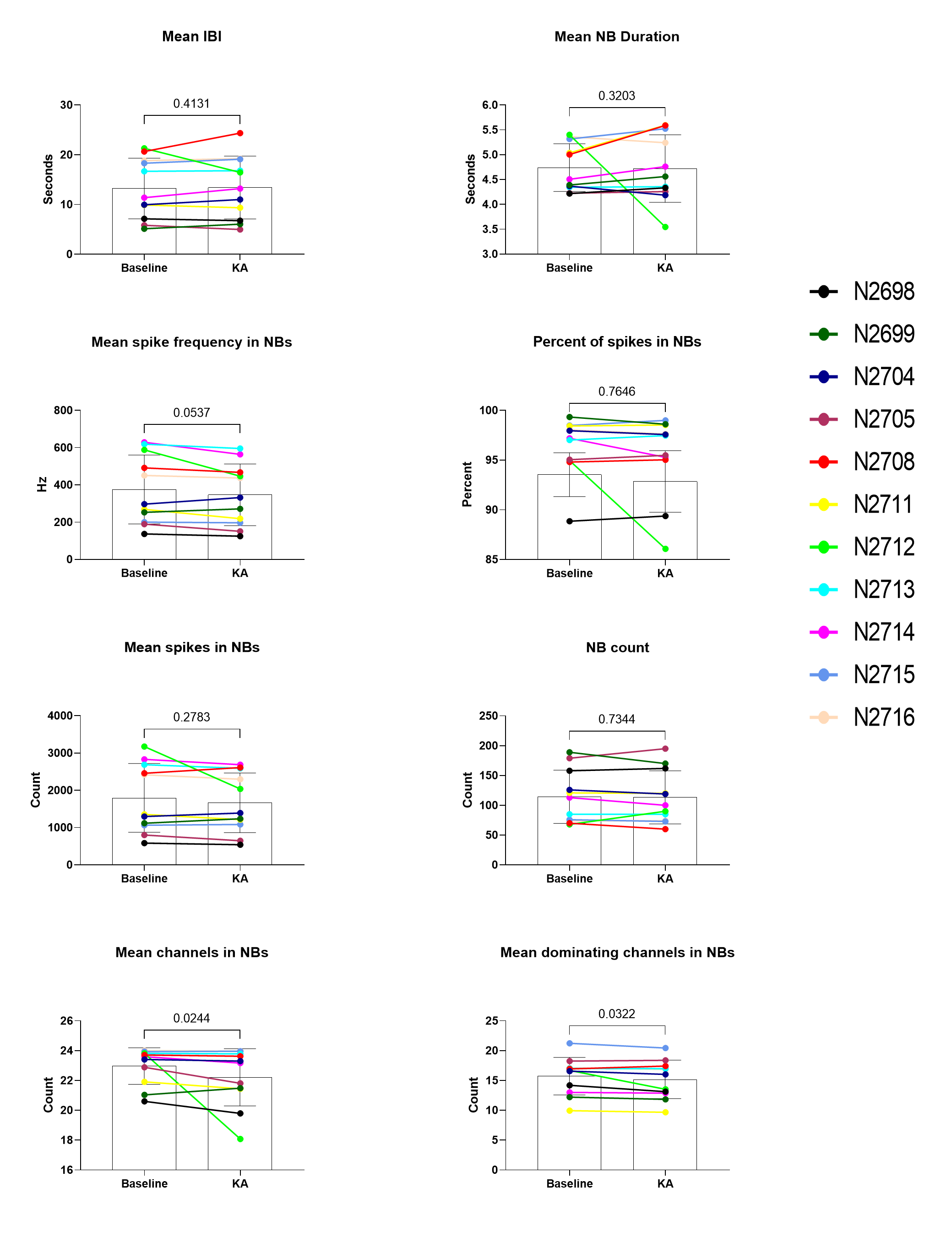

Supplement: Figure 4-1 — Pairwise comparisons of NB parameters before and after KA treatment in the distal A compartment. The color codes for the corresponding MEMO codes are shown on the side. The bar charts with whiskers present means and standard deviations. n = 11 for each pairwise comparison. p values are presented on top of each plot. The Wilcoxon matched-pairs signed-rank test was used. Download Figure 4-1, TIF file. [file eneuro-11-ENEURO.0035-24.2024-s003.tif]

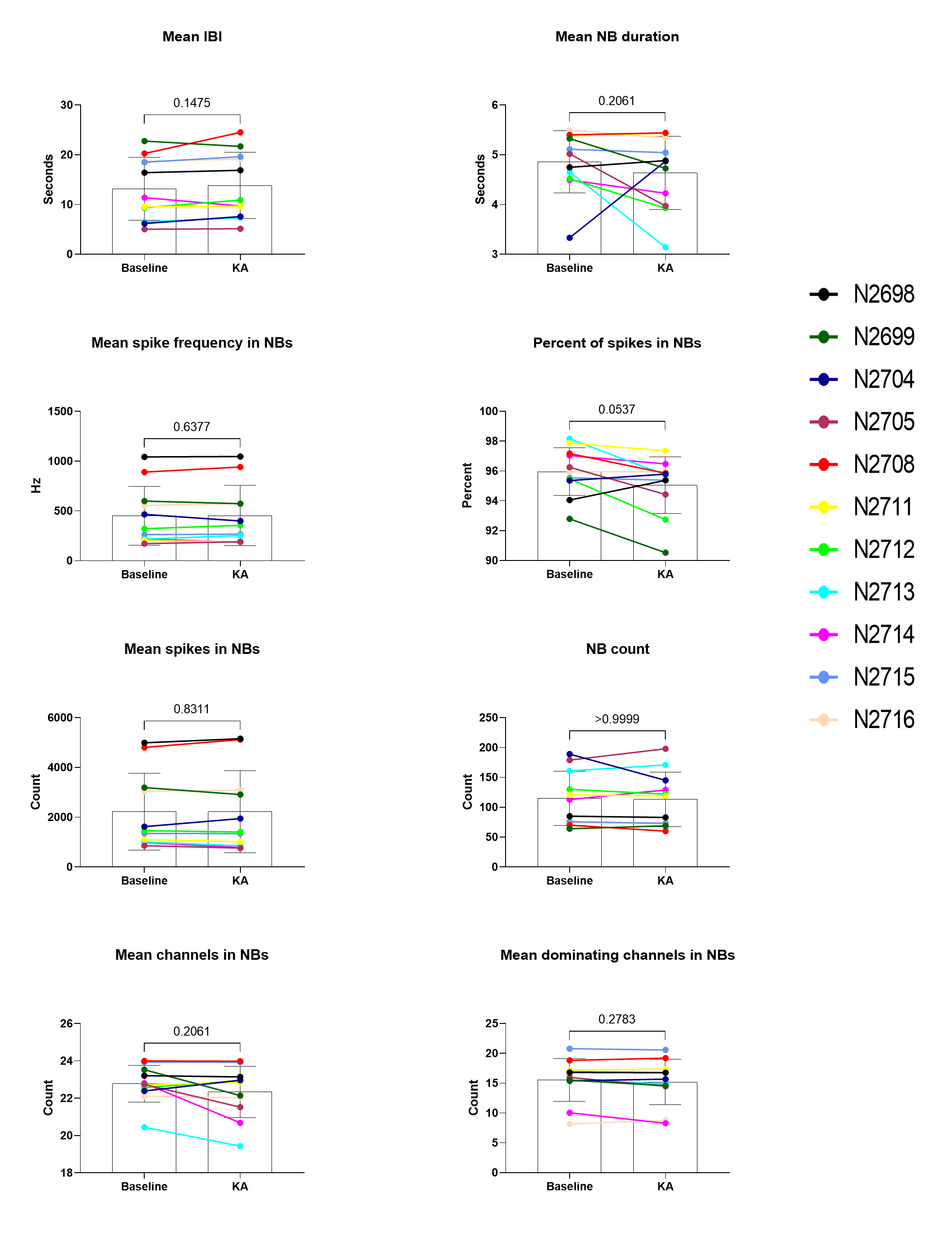

Supplement: Figure 4-2 — Pairwise comparisons of NB parameters before and after KA treatment in the distal B compartment. The color codes for the corresponding MEMO codes are shown on the side. The bar charts with whiskers present means and standard deviations. n = 11 for each pairwise comparison. p values are presented on top of each plot. The Wilcoxon matched-pairs signed-rank test was used. Download Figure 4-2, TIF file. [file eneuro-11-ENEURO.0035-24.2024-s004.tif]

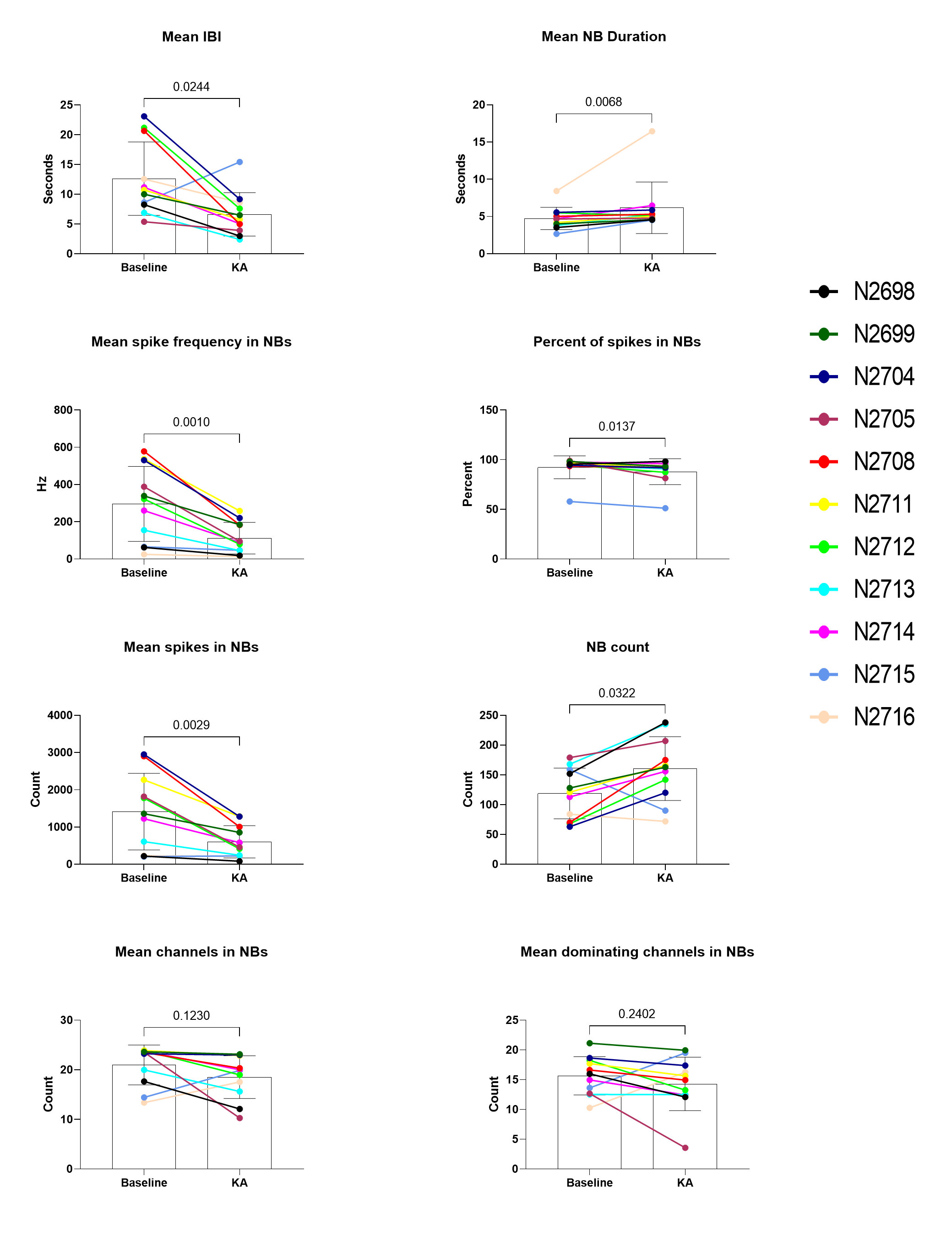

Supplement: Figure 4-3 — Pairwise comparisons of NB parameters before and after KA treatment in the proximal C compartment. The color codes for the corresponding MEMO codes are shown on the side. The bar charts with whiskers present means and standard deviations. n = 11 for each pairwise comparison. p values are presented on top of each plot. The Wilcoxon matched-pairs signed-rank test was used. Download Figure 4-3, TIF file. [file eneuro-11-ENEURO.0035-24.2024-s005.tif]

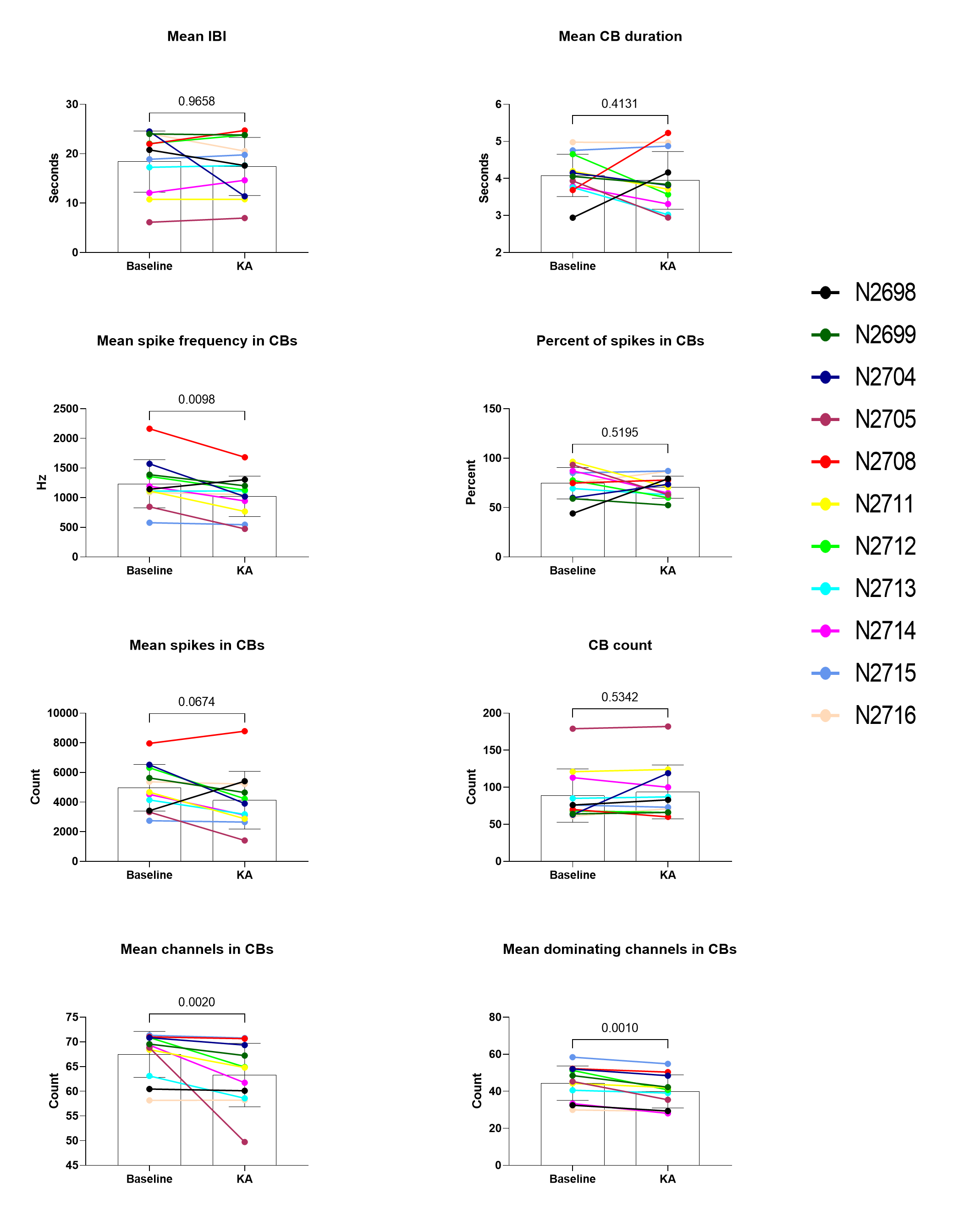

Supplement: Figure 4-4 — Pairwise comparisons of CB parameters before and after KA treatment at the circuit level. The color codes for the corresponding MEMO codes are shown on the side. The bar charts with whiskers present means and standard deviations. n = 11 for each pairwise comparison. p values are presented on top of each plot. The Wilcoxon matched-pairs signed-rank test was used. Download Figure 4-4, TIF file. [file eneuro-11-ENEURO.0035-24.2024-s006.tif]

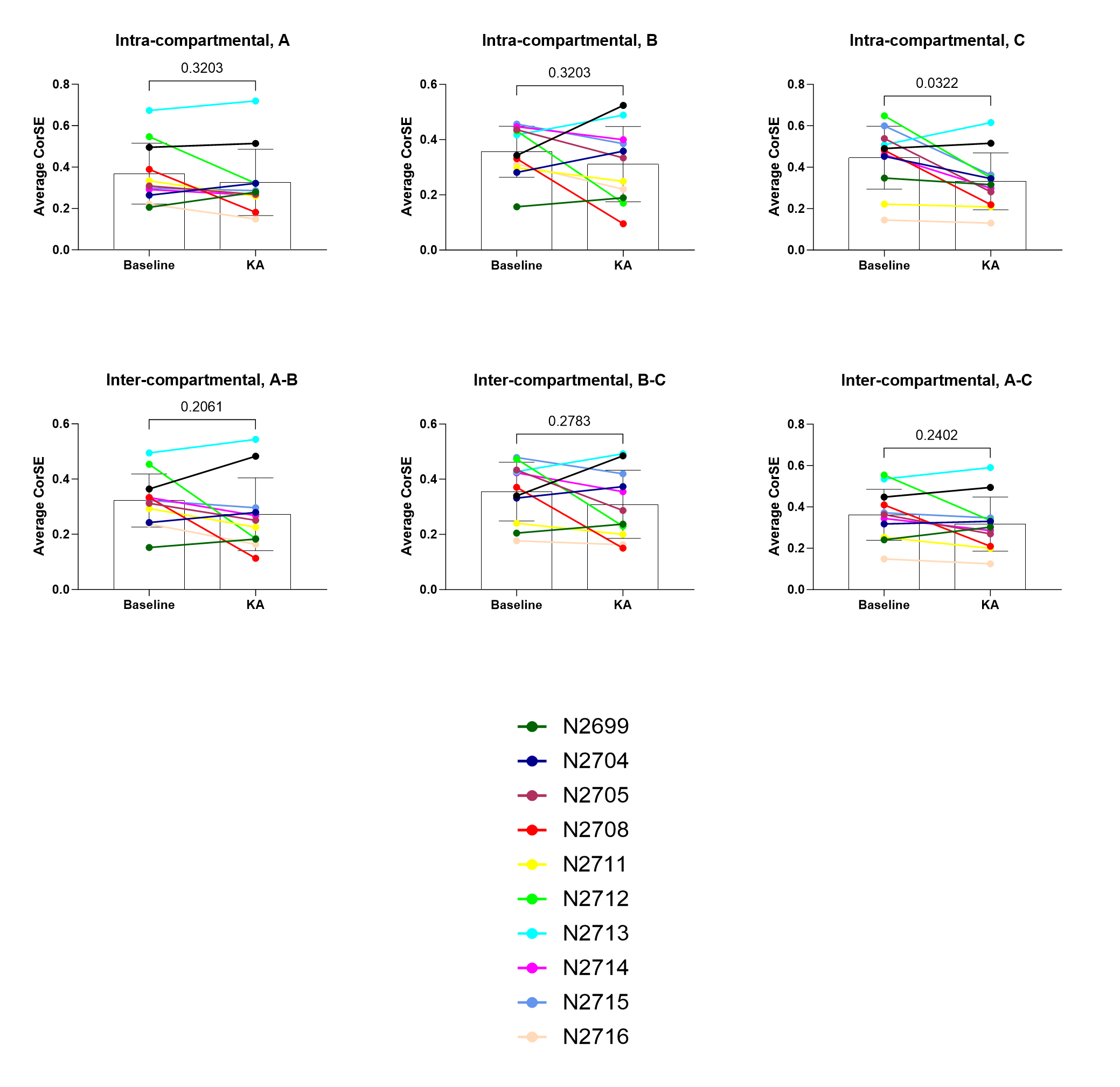

Supplement: Figure 6-1 — Pairwise comparisons of average intra- and intercompartmental CorSE values before and after KA treatment among MEMOs. The color codes for the corresponding MEMO codes are shown on the side. The bar charts with whiskers present means and standard deviations. n = 11 for each pairwise comparison. p values are presented on top of each plot. The Wilcoxon matched-pairs signed-rank test was used. Download Figure 6-1, TIF file. [file eneuro-11-ENEURO.0035-24.2024-s007.tif]

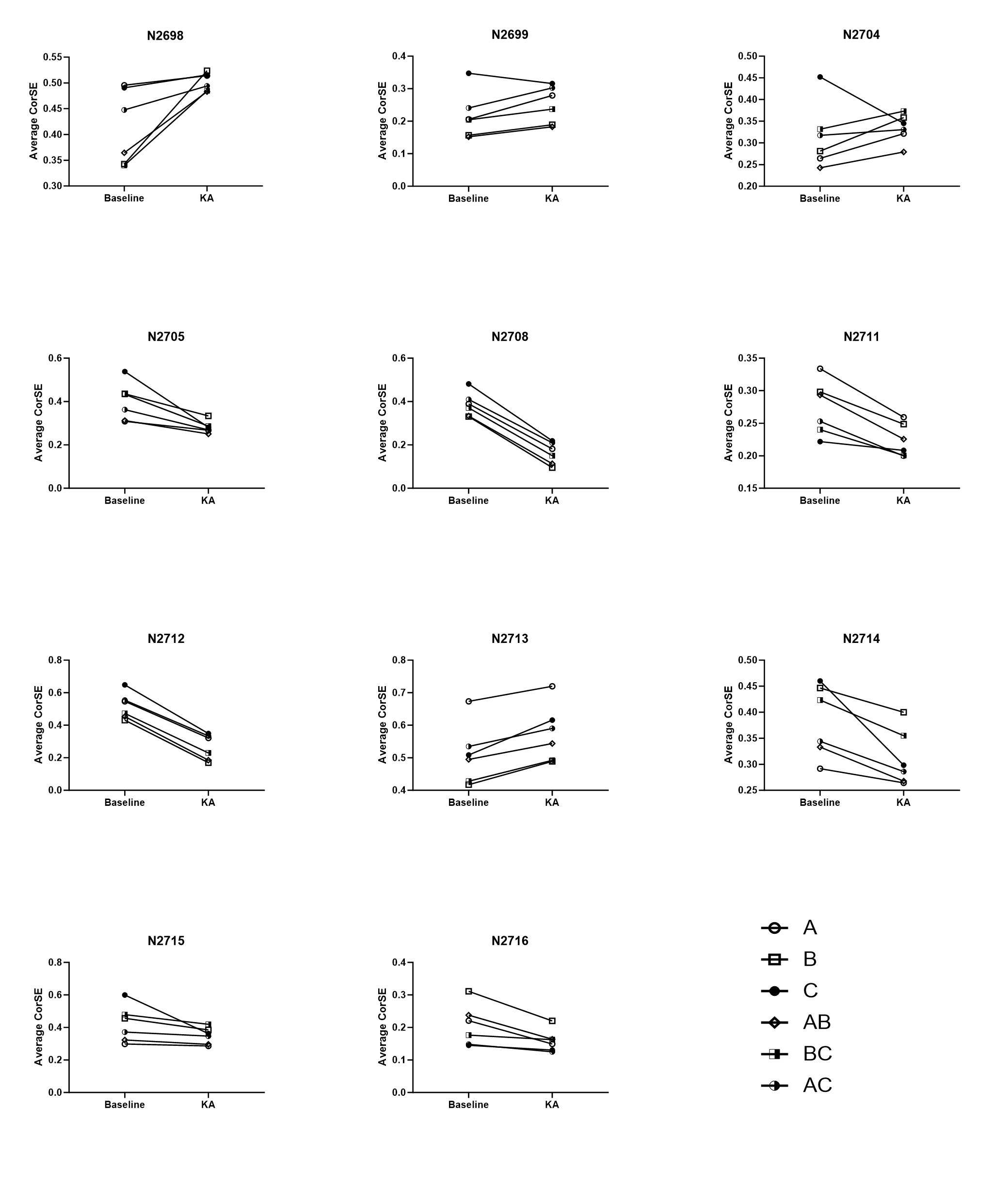

Supplement: Figure 6-2 — Pairwise comparisons of average intra- and intercompartmental CorSE values within each MEMO. Symbolic codes for corresponding connectivity are shown on the side. Download Figure 6-2, TIF file. [file eneuro-11-ENEURO.0035-24.2024-s008.tif]
