# Supplementary material for: Exploring Kainic Acid-Induced Alterations in Circular Tripartite Networks with Advanced Analysis Tools
Source: eNeuro. 2024 Jul 26;11(7):ENEURO.0035-24.2024. doi: 10.1523/ENEURO.0035-24.2024 (PMC11289587; doi:10.1523/ENEURO.0035-24.2024)
Supplement: Pseudocode — Download Pseudocode, DOCX file. [file eneuro-11-ENEURO.0035-24.2024-s009.docx]

PSEUDO-CODE

*For detection of Intermediate Circuitry Bursts (ICBs) and Circuitry Bursts (CBs) based on Network Burst (NBs) times from 3 compartments.*

def NETWORK_SUMMARY (A, B, C):

# Input:

NB times for each compartment are stored in *n*-by-2 matrices where the first column contains NBs’ start times and the second – to end times:

**A** – [*a*, 2] matrix for compartment A

**B** – [*b*, 2] matrix for compartment B

**C** – [*c*, 2] matrix for compartment C

# Function:

Step 1:

Arrange NBs' start and end times from all compartments into [*N*, 2] matrix **M** by stacking the matrices **A**, **B** and **C** on top of one another. *N*=*a*+*b*+*c* – total number of NBs from all compartments.

Step 2:

Vectorize the obtained matrix **M** into [2*N*, 1] column vector **T** by stacking the columns of the matrix **M** on top of one another.

Step 3:

Create a [2*N*, 1] column vector **O** of repeated compartment-specific numeric labels in accordance with NBs’ counts in each compartment. Hence, if we choose compartment A to be represented by 10, compartment B – by 100 and compartment C by 1000, we will get a vector with *a* tens, followed by *b* hundreds followed by *c* thousands followed by repeating the same entries multiplied by -1. Thus, *N* positive entries followed by *N* negative entries, which correspond to network bursts’ start and end times stored in vector **T.** For example, if *a*=4 *b*=3 *c*=2, the resulting vector **T** will look as below:

**T** = [ 10 10 10 10 100 100 100 1000 1000 -10 -10 -10 -10 -100 -100 -100 -1000 -1000 ]^T^

The compartment alphabetic labels and their corresponding numeric labels and numbers of NBs (*n*) are saved in table **L** for convenience.

Step 4:

Sort the vector **T** into **T*** in ascending order with tracking the shift of elements into index vector **I.**

Step 5:

Re-arrange vector **O** into vector **O*** in accordance with tracked index permutation stored in vector **I.**

Step 6:

Perform cumulative sum operation of vector **O*** and store the result as vector **S**.

# Output:

**S** – cumulative sum vector, which embeds flags for ICB and CB detection

**T*** – sorted vector of NBs’ start and end times from all compartments

def CIRCUITRY_ANALYSIS (**S**, **T***, **L, WELLS**):

# Input:

**S** – cumulative sum vector, which embeds flags for ICB and CB detection

**T*** – sorted vector of NBs’ start and end times from all compartments

**L** – the table of compartment alphabetic labels and their corresponding numeric labels

**WELLS** – a specification, which synchronization to check. For example, “*ABC*” implies full CB detection while “*AB*” means ICB detection between compartments A and B (ICB requires synchronization of 2 compartments, CB – all 3).

# Function:

Step 1:

Check if all compartments selected in **WELLS** exhibit NBs using table **L**.

Step 2:

Calculate the cumulative value **V** for synchronized compartments selected in **WELLS** using table **L**: for example, “*ABC*” selection for **WELLS** and **L** table with compartment A represented by 10, compartment B – by 100 and compartment C by 1000 imply **V** equal to 1110.

Step 3:

Find indices *s* of vector **S** which are equal to **V**. These correspond to the beginning of CBs in our case. The indices s+1 correspond to the end of CBs.*

Step 4:

Extract time values for CBs' starts (**STR**) and ends (**END**) from vector **T*** by taking its *s* and *s*+1 elements correspondingly.

Step 5 (optional):

Based on the detected CBs’ times apply enhancement steps.

# Output:

**STR** – vector of CB start times

**END** – vector of CB end times

* For ICB case possible **V** values are 1010, 1100 and 110. We additionally checked nearest *s*-1 and *s*+1 elements for CB-corresponding **V** value 1110. If it was detected, the index was discarded as we decided to discard ICBs adjacent to CBs from our analysis.
