# Supplementary material for: Exploring Kainic Acid-Induced Alterations in Circular Tripartite Networks with Advanced Analysis Tools
Source: eNeuro. 2024 Jul 26;11(7):ENEURO.0035-24.2024. doi: 10.1523/ENEURO.0035-24.2024 (PMC11289587; doi:10.1523/ENEURO.0035-24.2024)
Supplement: Table 2-1 — The top row shows the MEMO platform identification code. The table lists the numbers of detected synchronous activity patterns among 11 MEMOs after KA exposure. ICB labels are created from the two letters of the corresponding involved compartment labels. The ICBs that were associated with the CBs were not considered and hence were not included in the table. ICB refers to intermediate circuitry burst, and CB refers to circuitry burst. Download Table 2-1, DOCX file. [file eneuro-11-ENEURO.0035-24.2024-s002.docx]

|  | N2698 | N2699 | N2704 | N2705 | N2708 | N2711 | N2712 | N2713 | N2714 | N2715 | N2716 |
| --- | --- | --- | --- | --- | --- | --- | --- | --- | --- | --- | --- |
| NBs A | 162 | 170 | 119 | 195 | 60 | 120 | 90 | 85 | 100 | 73 | 74 |
| NBs B | 83 | 69 | 145 | 198 | 60 | 119 | 122 | 171 | 129 | 73 | 74 |
| NBs C | 238 | 163 | 120 | 207 | 175 | 166 | 142 | 235 | 156 | 90 | 72 |
| ICBs AB | 0 | 0 | 0 | 24 | 0 | 0 | 0 | 0 | 0 | 0 | 3 |
| ICBs BC | 1 | 0 | 2 | 8 | 0 | 0 | 39 | 84 | 29 | 0 | 0 |
| ICBs AC | 82 | 104 | 0 | 0 | 0 | 5 | 24 | 0 | 1 | 0 | 0 |
| ICBs total | 83 | 104 | 2 | 32 | 0 | 5 | 63 | 84 | 30 | 0 | 3 |
| CBs | 83 | 66 | 119 | 182 | 60 | 124 | 66 | 87 | 100 | 73 | 71 |

Table 2-1. The top row shows the MEMO platform identification code. The table lists the numbers of detected synchronous activity patterns among 11 MEMOs after KA exposure. ICB labels are created from the two letters of the corresponding involved compartment labels. The ICBs that were associated with the CBs were not considered and hence were not included in the table. ICB refers to intermediate circuitry burst, and CB refers to circuitry burst.
